# Evaluation of Canonical Inflammasome Activation in Human Monocytes by Imaging Flow Cytometry

**DOI:** 10.3389/fimmu.2019.01284

**Published:** 2019-06-04

**Authors:** Silvia Lucena Lage, Venina Marcela Dominical, Chun-Shu Wong, Irini Sereti

**Affiliations:** ^1^National Institute of Allergy and Infectious Diseases, Bethesda, MD, United States; ^2^Flow Cytometry Core Facility, National Heart, Lung, and Blood Institute, Bethesda, MD, United States

**Keywords:** inflammasome, ASC speck, multispectral imaging flow cytometry (MIFC), monocytes, caspase-1

## Abstract

Canonical inflammasome activation is a tightly regulated process that has been implicated in a broad spectrum of inflammatory disorders. Inflammasome formation requires assembly of a cytosolic sensor protein with the adapter, ASC (apoptosis-associated speck-like protein containing a caspase activating and recruitment domain). Once formed, this multimeric protein structure allows for the activation of caspase-1, responsible for IL-1ß/IL-18 release. During this process, cytoplasmic dispersed ASC molecules cluster in one condensed micrometric-sized complex named ASC “speck,” which is traditionally assessed by fluorescence microscopy and widely accepted as a readout for canonical inflammasome activation. However, equally reliable but less time-consuming quantitative methods have emerged as a significant need in order to improve clinical assessment of inflammasome-related conditions. Multispectral imaging flow cytometry (MIFC) combines the qualitative power of fluorescence microscopy with high throughput capabilities and multiplexing potential of flow cytometry into one single system. Here we explored the optimal imaging-based tools to measure ASC speck formation via imaging flow cytometry by using peripheral blood mononuclear cells (PBMCs) stimulated with the NLRP3 agonist Nigericin, as a positive control. We demonstrate that this technique is also able to detect the distribution of active caspase-1 within the ASC aggregates by incubating cells with FAM-FLICA^TM^, a fluorochrome inhibitor of caspase-1. By applying these tools in PBMCs from patients with distinct inflammatory disorders we demonstrate that MIFC is able to assess canonical inflammasome activation in a quantitative and statistically robust manner in clinically relevant samples. Therefore, we propose that accurate assessment of specks by MIFC could help guide preventive or therapeutic strategies in an array of human inflammatory diseases in which inflammasomes play an important role.

## Introduction

Inflammasomes are cytosolic protein aggregates that are assembled in order to coordinate distinct immune responses to infectious agents or physiological anomalies (1, 2). Despite their contribution to the immune response against pathogens, dysregulation of inflammasomes has been implicated in a broad spectrum of mammalian diseases such as cancer (3), cardiovascular and neurodegenerative disorders (4–6), autoinflammatory syndromes (7), diabetes (8), and multiple sclerosis (9). Inflammasomes are formed when members of the NLR (nucleotide-binding oligomerization domain (NOD)-like receptor) family or the HIN200 (hematopoietic interferon-inducible nuclear antigens with 200 amino-acid repeats) family, sense either microbial components or host-derived molecules (2). Upon activation, these sensors oligomerize and recruit an adaptor protein, ASC (apoptosis-associated speck-like protein containing a caspase activating and recruitment domain or CARD). ASC is comprised of two conserved domains: a pyrin domain (PYD) and a CARD domain. Some inflammasome sensors, such as NLRP1 (NOD, leucine rich repeat (LRR)-, PYD-containing 1) (1, 10), NLRP3 (11–14), AIM2 (absent in melanoma 2) (15) and the Pyrin protein (16), also possess a PYD effector domain, and thus can recruit ASC through homotypic PYD-PYD interactions. ASC, then assembles into large helical fibrils through oligomerization with successive ASC proteins via their PYD domains, in a prion-like fashion (17, 18). Inflammatory caspases are recruited to ASC filaments via a CARD domain interacting with the free CARD domain of the ASC fibrils. These effector caspases are caspase-1, −4, and −5 in humans and caspase-1, −11, and −12 in mice, although caspase-1 is the most commonly inflammasome-associated caspase (1, 19).

Finally, filaments, containing sensor proteins and an excess of ASC-caspase-1 molecules, interact with each other, thus forming a ring-like perinuclear complex called ASC “speck” (20). Once recruited to the ASC focus, procaspase-1 molecules undergo autocatalytic cleavage which allows their activation into a mature form that is now able to cleave and release the pro-inflammatory cytokines: IL-1β and IL-18, previously stored in the cytoplasm in their inactivated forms (1, 21). Inflammasome assembly is also accompanied by gasdermin D (GSDMD) activation, which in its turn promotes a pro-inflammatory form of cell death called pyroptosis (22). The inflammasome sensor NLRC4 (NOD-, LRR-, CARD-containing 4), contains a CARD effector domain, which allows for NLRC4 to either directly recruit pro-caspase-1 or use ASC as a linker protein via CARD–CARD interactions (23, 24). Although recruitment of caspase-1 by NLRC4, without ASC, is sufficient to induce GSDMD-mediated pyroptosis, it does not rely on IL-1β/IL-18 cytokine maturation and secretion (25). It has been suggested that ASC clustering brings caspase-1 molecules in close proximity, which is crucial to drive caspase-1 autoproteolysis into the active heterodimer able to cleave IL-1β and IL-18 into their mature forms, highlighting the importance of ASC speck formation for cytokine secretion downstream of distinct inflammasome sensors.

Based on these data, ASC aggregates are considered the hallmark of canonical inflammasome activation and assessment of ASC speck formation is one of the most commonly used approaches to study inflammasomes. ASC puncta has been traditionally assessed by fluorescence microscopy in many studies, where it is possible to observe ASC redistribution in the cytoplasm, from a dispersed form in resting cells to a single condensed complex upon inflammasome activation (20, 26). Analysis in superresolution microscope demonstrates that ASC aggregates can vary from 0.5 to 1 um in size and may contain inflammasome sensors and caspase-1 in the core of the complex (27). Therefore, given the importance of inflammasomes in the pathogenesis of numerous infectious diseases and inflammatory disorders, delineating a faster and accurate method that could be used for diagnosis, prognosis or response to treatment of these conditions has emerged as a critical need. A faster method to measure ASC oligomerization by flow cytometry was recently reported. Authors were able to discriminate between resting vs. inflammasome-activated cells, based on the fact that spread ASC gave a low signal pulse with a width corresponding to the cell size, while ASC clustering in one speck resulted in a higher signal pulse with a smaller width (28, 29).

Here, we will extend on these approaches to detect ASC specks by describing a method that combines the speed and statistical power of flow cytometry with the spatial information achieved by fluorescence microscopy. In recent years, measurement of distinct image-based features by Multispectral imaging flow cytometry (MIFC) has been gaining popularity due to its high throughput capabilities and multiplexing potential. MIFC associates the features of conventional flow cytometry and fluorescence microscopy in a single system. It incorporates bright and dark field images of single cells in flow and allows the digitalization of thousands of cells per minute while measuring fluorescence as a conventional flow cytometer. MIFC is an emerging technique that improves the assessment of important cellular processes, like autophagy (30), cell death (31) and neutrophil-mediated NETosis (32). Here, we will outline the best methodological strategies that can quantify inflammasome activation by measuring ASC specks in human monocytes via imaging flow cytometry ImageStream (MilliporeSigma). Importanty, by showing increased active caspase-1^+^speck formation in patient samples, in comparison with healthy individuals, our findings support the feasibility of using MIFC as a powerful approach to identify inflammasome aggregates as biomarkers or therapeutic targets in inflammasome-related disorders.

## Materials and Methods

### Ethics Statement

Healthy volunteers blood samples were obtained on the NCI, NIH IRB-approved protocol 99-CC-0168 and de-identified prior to distribution. Patient samples were collected under the following IRB-approved protocols: Immune Reconstitution Syndrome in HIV-Infected Patients Taking Antiretroviral Therapy, NCT00286767; Gene Expression in HIV and Tuberculosis Co-infection, NCT01611402; PET Imaging and Lymph Node Assessment of IRIS in People With AIDS, NCT02147405. All subjects gave written informed consent in accordance with the Declaration of Helsinki.

### Cell Culture

Cryopreserved ficoll-isolated peripheral blood mononuclear cells (PBMCs) from healthy individuals or patients were thawed and resuspended in RPMI-1640 media (Corning, NY, USA) supplemented with 10% heat-inactivated human AB serum (Gemini Bio-Products, West Sacramento, CA, USA) and 0.05% benzonase (MilliporeSigma, Massachusetts, USA). Cells were rested for 1 h at 37°C and 5% CO_2._ They were subsequently plated at 10^6^ cells/well in round bottom 96-well plates (Corning Costar^TM^, MilliporeSigma, Massachusetts, USA).

### Treatment and Labeling

To achieve canonical NLRP3 inflammasome activation, PBMCs from healthy subjects were first primed with the TLR2 ligand, Pam3CSK4 (Invivogen, San Diego, California, USA) (0.5 μg/mL, 3 h), in order to upregulate the expression of inflammasome-related molecules. Cells were further stimulated with 3.5 μM of Nigericin (1 h) (Invivogen) to promote signal two for NLRP3 inflammasome activation. In order to assess the presence of speck-associated active caspase-1, cells were incubated with the fluorochrome inhibitor of caspase-1 (FAM-FLICA^TM^, Immunochemistry technologies (ICT), Bloomington, MN, USA) following the manufacturer's instructions. Briefly, a vial of FLICA (previously reconstituted with 50 μl of DMSO to give a stock of 150x) was diluted 1:5 with PBS to achieve a working concentration of 30x. Control and stimulated cells were resuspended with 1x FLICA reagent in complete media and incubated for 50 min at 37°C, to allow for binding of FLICA to activated caspases. Finally, cells were washed twice with FLICA wash buffer to allow any unbound reagent to diffuse out of the cells. Cells were then incubated with Live/Dead Fixable AQUA Dead Cells Stain (Thermo Fisher, Waltham, Massachusetts, USA) for 15 min at room temperature (RT), washed with PBS and stained in PBS + 1% BSA with the following fluorochrome-conjugated antibodies for monocyte phenotyping: anti-CD14 BV605 (Clone:M5E2) and anti-CD3 PE (Clone:HIT3a) from BioLegend, San Diego, CA, USA; anti-CD20 PE (Clone:2H7), anti-CD19 PE (Clone:SJ25C1), anti-CD2 PE (Clone:RPA-2.10), from eBioscience, San Diego, CA, USA; anti-CD56 PE (Clone: B159 (RUO) from BD Biosciences, Franklin Lakes, New Jersey, USA. Stained cells were fixed and permeabilized with Cytofix/Cytoperm (BD Biosciences) for 1 h at RT and kept overnight at 4°C. After permeabilization, cells were stained for 1 h at RT for intracellular ASC, with anti-ASC/TMS1 AF647 antibody from Novus Biologicals, Littleton, CO, USA. For nuclear staining, DAPI (Life Technology, Pittsburgh, PA, USA) was added to the samples at 300 nM in PBS for 5 min and washed away by centrifugation. Samples were resuspended in 50 μL of PBS+1%BSA and kept at 4°C. Alternatively, patient cells proceeded to FLICA incubation followed by staining without any further treatment. The addition of the anti-HLA-DR BV421 [Clone: G46-6 (RUO)] from BD Biosciences, Franklin Lakes, New Jersey, USA) in the extracelular staining, instead of DAPI staining, was used here to better identify circulating monocytes from patients as HLA-DR^+^DUMP^−^ population. Cells were fixed, permeabilized and stained with anti-ASC/TMS1 as described above, then resuspended in 50 μL of PBS+1%BSA and kept at 4°C.

### Image Acquisition

Approximately 40,000 focused cells for each sample were acquired using a 12 channel Amnis ImageStreamX Mark II (MilliporeSigma) imaging flow cytometer equipped with the 405 nm, 488 nmand 642 nm lasers. Channel 2 (FLICA), Channel 3 was used as a DUMP channel to exclude other cell types than monocytes (CD2, CD3, CD19, CD20, CD56, CD66b–“DUMP”-PE), Channel 7 (DAPI or HLA-DR), Channel 8 (Live/Dead-AQUA), Channel 10 (CD14-BV605) and Channel 11 (ASC-AF647) were selected. For brightfield and SSC detection (785 nm laser), channels 1/9 and 12 were used, respectively. Samples were acquired at 60X magnification and low speed/high resolution. The integrated software INSPIRE (MilliporeSigma) was used for data collection. Single color compensation controls were also acquired. Stimulated cells stained with FAM-FLICA^TM^ should be utilized as a FLICA positive single stained compensation control. DAPI-stained cells were also used to compensate Channel 7.

### Image Analysis Using IDEAS

Images were analyzed using image-based algorithms in the ImageStream Data Exploration and Analysis Software (IDEAS 6.2.64.0, MilliporeSigma) as follows. Single color controls were used to calculate a spectral crosstalk matrix, which was applied to each of the files for spectral compensation of the fluorescence in the detection channels. In order to analyze the compensated data files, it is important to highlight the concept of “Features” and “Masks.” Features are algorithms created by the software that contains the morphological measurements obtained from an image. In other words, features are image-based parameters, like size, shape, and texture that allow users to discriminate cells based on their characteristics. It is possible to apply those features on the entire cell or on a specific cell compartment (e.g., nucleus, membrane, cytoplasm) by using a mask to spatially distinguish the region of interest within the cell in which the feature will be applied. Eight feature categories are available for users in IDEAS: “size,” “location,” “shape,” “texture,” “signal strength,” “comparison,” “system and combined” (via IDEAS user's manual). By default, IDEAS presents specific application wizards (“apoptosis wizard,” “cell cycle—mitosis,” “co-localization,” “internalization,” “nuclear localization,” “shape change” and “spot wizard”) that explore features including: “similarity,” “spot count,” “compactness,” “fluorescence intensity,” “aspect ratio,” etc. When a specific application wizard is not appropriate, however, users can choose between creating custom features or applying the “Feature Finder Wizard.” The latter is most appropriate for choosing the best feature for the intended analysis, when it is unclear which feature will discriminate better between phenotypes. In this work, in order to find the feature that would better discriminate ASC speck formation, we applied the “Feature Finder Wizard” on a merged file of nigericin-stimulated cells, when we expected to find the majority of positive events for Specks- with a control file with RPMI-cultured cells expected to have diffuse ASC throughout the cytoplasm. The description of Features and Masks used in this study was obtained from the IDEAS User's Manual guide.

### Statistical Analyses

Statistical analyses were performed using non-parametric Mann-Whitney test in GraphPad Prism 4 software (GraphPad, USA). Data are presented as median with interquartile ranges. Differences between groups were considered significant when *p* < 0.05.

## Results

### Identification of Monocytes

**Figure 1:**
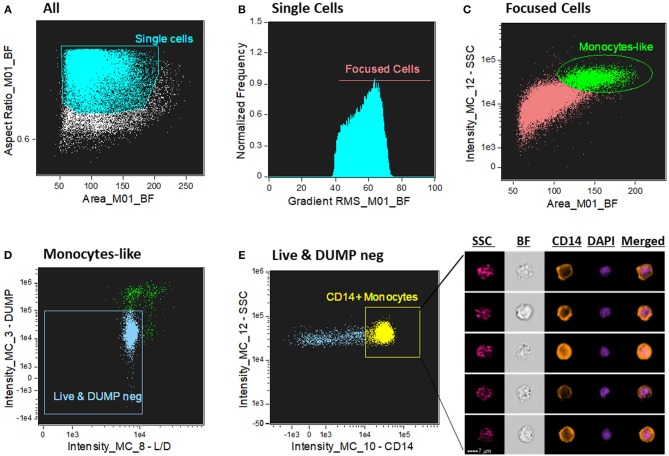
Gating strategy for CD14^+^ monocyte identification. **(A)** Dot-plot of Area vs. aspect ratio of BF (channel 1). Single cells present higher aspect ratio and mid-range area. The boundaries of the single cells gate were determined by looking at the imagery in the gallery area. **(B)** Histogram of Gradient RMS of the ch1 (BF) to select cells in good focus. Focused cells present greater difference with the background and consequently higher score in this texture feature. **(C)** Identification of monocytes by Area of the BF (ch1) vs. Side Scatter (SSC—ch12); monocytes present higher SSC values in the PBMC fraction compared to lymphocytes. **(D)** 2-parameter dot-plot fluorescence of Live/Dead (L/D—channel 8) cell marker (X axis) and DUMP channel (channel 3–Y axis). Double negative events for these two markers exclude dead cells and non-monocytes (CD3^+^, CD56^+^, CD19^+^, etc). **(E)** 2 parameter dot-plot of fluorescence intensity of CD14 vs. SSC. Monocytes are identified by selecting CD14^+^ cells. On the right side of the graph, representative images of CD14+ monocytes were selected, showing, respectively: SSC (side scatter—granularity), BF (brightfield), CD14 (orange color), DAPI (purple color) and composite image of the two fluorescence dyes (CD14 and DAPI). Intensity fluorescence was augmented for the purpose of imagery, not affecting results and statistics.

We sought to determine human CD14^+^ monocytes as our population of interest. First, single cells were gated from cell aggregates and debris by using a scatter plot of the brightfield of area vs. aspect ratio (Figure 1A). Single cells have an intermediate area value and aspect ratio scores close to 1. Aspect Ratio, which is the minor axis divided by the major axis of an object, measures the elongation of an object where round events will have Aspect Ratio values close to 1, which is where single cells are located. Next, from the single cells gate population, the cells in best focus were identified in the histogram of gradient RMS of the brightfield channel (channel 1). This texture feature allows to determine overall focus quality of the objects by using the average gradient of a pixel normalized for variations in intensity levels, i.e., bigger changes of pixel values in the image correspond to sharper imagery (more contrast with the background -Figure 1B). From the focused single cells population, a classical flow cytometry dot-plot of Area vs. Side Scatter (SSC) was applied to identify, based on size and granularity, monocyte-like cells from the PBMC suspension. Because monocytes are bigger and more complex (with more granules) compared to lymphocytes, we selected a region with those characteristics to further refine and characterize these cells (Figure 1C). From there, live (AQUA channel 8) and DUMP negative cells (PE –channel 3) were further refined for monocytes identification. Here, the dump channel was used to gate out any non-monocytic remaining cells to avoid other cell type contamination (cells positive for one of the following antigens: CD2, CD3, CD19, CD20, CD56, CD66b—PE labeled, were excluded) (Figure 1D). Lastly, the final characterization of monocytes was made by a CD14 fluorescence intensity vs. SSC dot-plot (Figure 1E), where cells positive for CD14 were gated for downstream analysis of the ASC expression pattern.

### Screening the Best “Features” to Discriminate Diffuse and Clustered ASC Staining

**Figure 2:**
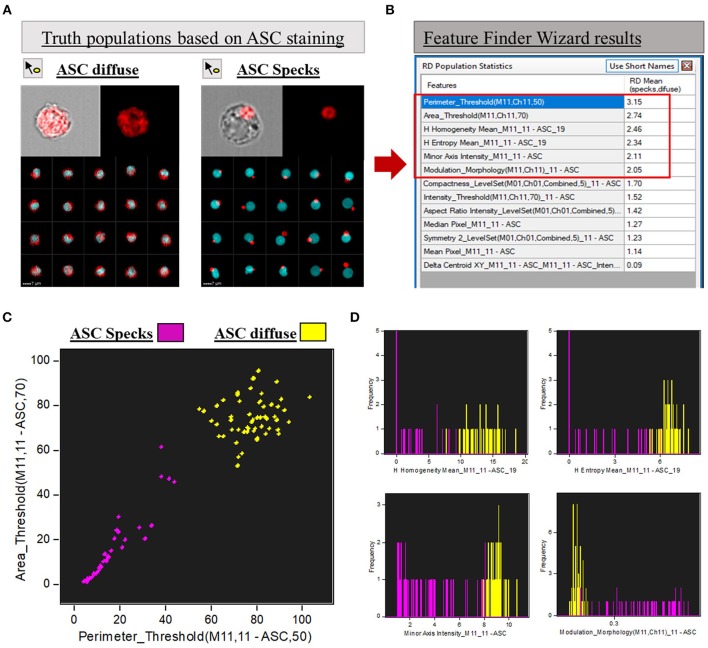
Feature Finder Wizard results for ASC speck discrimination. **(A)** Representative imagery in the gallery area of Diffuse vs. ASC speck phenotypes assigned as “truth populations” for Feature Finder screening. **(B)** “Feature Finder Wizard Results” table of contents ranking the top 13 positions for speck discrimination. Red square highlighted the Top 6 positions due to Rd scores higher than 2. **(C)** Representative Dot-plot of the top 2 features suggested by Feature Finder: Area_Threshould(M11,11-ASC70) vs. Perimeter_Threshould(M11,11-ASC,50) showing the separation of the tow assigned “truth populations.” **(D)** Histograms of the “truth populations” according with the other ranking Features that achieved Rd scores higher than 2, as follows: H Homogeneity Mean_M11,11-ASC19, H Entropy Mean_M11,11-ASC19, Minor Axis Intensity_M11,11-ASC, Modulation_Morphology(M11,Ch11)_11-ASC.

Following the “Feature Finder Wizard” instructions, we created a gallery of two “truth populations” of cells that represent the phenotypical differences we wanted to discriminate: cells with cytoplasmic diffuse ASC and cells containing aggregated ASC (ASC speck). By using the tagging tool icon we hand-selected at least 50 images of these two distinct groups inside the CD14^+^ ASC^+^ population. Images of both groups are represented in Figure 2A. Next, we selected channel 11 (ASC channel) and all the 8 feature categories we wanted to explore to evaluate their ability to separate the assigned truth populations. The feature categories in IDEAS are size, shape, location, signal strength, texture, comparison, combined and all. By selecting these features, the software will determine the best feature that can separate the two phenotypes. For example, size features will separate the data based on the size (in microns) of the mask in the channel selected. Area, height, length, perimeter, minor or major axis are all examples of features that fall under this category; while shape features are based on mask shape of the object (e.g., aspect ratio, symmetry, circularity, compactness, etc.). Signal strength is measured in pixel values, and intensity is the feature used in this category. Texture features measure pixel or regional variation, thus indicating the granularity or complexity of an image. Included under this category are: H texture features, contrast, modulation, spot count, and standard deviation features. For further details and description of the features in IDEAS, please refer to IDEAS user's manual in the customer portal in the Amnis MilliporeSigma website.

To calculate the best statistical separation between “truth populations” for each feature, Feature finder uses Fisher's discriminant ratio (Rd), which is the difference in the means divided by the sum of the standard deviations for the two populations as follows: Fisher's discriminant ratio (Rd): Rd = Mean1 - Mean2 / StdDev1 + StdDev2. Where 1 and 2 refer to the two “truth populations” assigned by the operator. The highest the Rd value, the better the separation achieved by the feature according with the algorithm. Therefore, Rd numbers help the operator to screen the best features to analyze the data among all the possibilities offered by the software. The “Feature Finder Wizard” ranks the top 13 Rd score features calculated for our “truth populations,” listed in Figure 2B (box in the right). Usually, Rd scores above 1.5 are widely accepted as a good indicative that the feature can discriminate well the two groups tested. In this work, we chose the top 6 features (Rd score >2) to represent graphically the separation of the two phenotypes when the respective features were applied (Figures 2C,D). A brief description of those features can be found in Table 1.

**Table 1:** Description of the features ranked in the top 6 positions for Speck discrimination (please, refer to Figures 2, 3 for more details).

**Feature**	**Description**	**ASC phenotype *(Specks compared to diffuse signal)***
Perimeter	Measures the boundary length of the mask in the number of microns. ^*^Threshold mask is used to exclude pixels, based on a percentage of the range of intensity values as defined by the starting mask.	Specks present lower perimeters—lower scores
Area	The number of microns squared in a mask is equal to the Area. The number of pixels is converted to μm^2^. Note that 1 pixel = 0.25 μm^2^.	Specks present lower areas due to clustering of signal
H Features	The user defines the texture grain by assigning a granularity value. For very fine textures, this value is small (1–3 pixels), while for very coarse textures, it is large (>10). In the IDEAS default template, the granularity value is 5. H features have value for distinguishing cellular texture when used individually, images often contain a mixture of different textures at different grains. Therefore, these features are most powerful when combined. **H Homogeneity mean** Homogeneity is a measure of how close pixels value are, if pixel pairs are the same or similar then a high homogeneity value is given. If there are very few pixels with the same value, then a lower homogeneity score is generated. Images with high homogeneity would look very uniform and lack texture. Homogeneity is the inverse of contrast. **H Entropy Mean** Entropy is a measure of high intensity concentration in the cell. However, this feature relates to the randomness of the intensities in the image. Images that have distinct areas of intensity concentration are less random and thus, have low entropy. Images that have a range of equally likely intensity pairings have less distinct intensity concentrations, and correspondingly, have higher entropy.	Because Specks are less homogeneous and have distinct areas of intensity concentration, thus presenting low scores for H homogeneity mean and H entropy mean, respectively
Minor Axis Intensity	This feature is the narrowest dimension of the ellipse of best fit and is intensity weighted.	Specks present lower dimension of the minor axis of the ASC intensity
Modulation	The Modulation feature measures the intensity range of an image, normalized between 0 and 1. The formula is: Modulation = Max Pixel—Min Pixel/Max Pixel + Min Pixel, it quantifies image quality and characterize contrast and texture in cells. ^*^Morphology mask includes all pixels within the outermost image contour. This mask, which is used in fluorescence images, is best used for calculating the values of overall shape-based features.	Specks present higher modulation scores due to higher contrast of ASC fluorescence with background

* *Description of the masks used in the respective feature. The description of the features and masks presented here can be found in the IDEAS User's Manual by assessing Amnis customer portal website*.

### Association of “Modulation” and “Area” Better Identify ASC Specks

**Figure 3:**
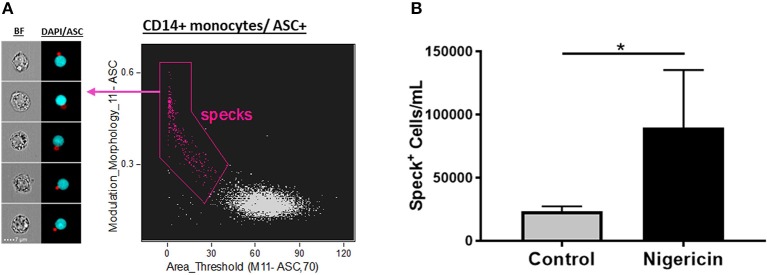
Quantification of ASC specks in nigericin stimulated-CD14^+^ monocytes. **(A)** Dot-plot of Area_Threshould(M11,11-ASC70) vs. Modulation_Morphology(M11,Ch11)_11-ASC inside CD14^+^ monocyte gate better identifies ASC specks in pam3csk4 (0.5 ug/mL, 3 h)- primed and nigericin (3.5 uM, 1 h)-stimulated PBMCs. On the left side of the graph, representative images of Speck^+^CD14^+^ monocytes were selected, showing, respectively: BF (brightfield) and composite image of the two fluorescences (ASC and DAPI). Intensity fluorescence was augmented for the purpose of imagery, not affecting results and statistics. **(B)** Quantification of Speck^+^ monocytes/mL in control vs. Nigericin-stimulated cultures after Area_Threshould(M11,11-ASC70) vs. Modulation_Morphology(M11,Ch11)_11-ASC dot-plot was applied in all data set. Numbers are the means ± SEM (*n* = 5). ^*^P < 0.05 when Mann-Whitney test was applied.

Next, to validate the feature finder results, we applied these top 6 features to our data set in order to evaluate their ability to identify Speck^+^ vs. Speck^−^ cells inside the ASC^+^ monocyte gate. All the top 6 features successfully discriminated our phenotype when applied individually or two of those features combined in a dot-plot (data not shown, *p* < 0.05 nigericin vs. control). Interestingly, by validating our gates through directly visually checking the correspondent images in the gallery, we observed that the combination of “Modulation” and “Area,” a texture and size feature types, respectively, was slightly more restrictive to determine the specks, by gating out “false positives” events and consequently giving more reliable counts (Figure 3A). We, then, applied the template analysis to all data files (Nigericin-stimulated or unstimulated cells) to extract the statistical results of number of Specks/mL obtained from the Modulation vs. Area dot-plot. As shown in Figure 3B, Nigericin-stimulated cultures showed higher number of monocytes presenting ASC speck formation in comparison with controls (*n* = 5, *p* = 0.0134, via *t*-test). Our data confirm that imaging flow cytometry provides an efficient and reliable method to quantify inflammasome activation by showing that different features in the IDEAS software are able to discriminate between cells with cytoplasmic diffuse ASC and cells with ASC aggregates. Additionally, we verified that applying size and texture features together (in this study Area vs. Modulation) did a superior job compared to any of the suggested 6 features when by itself (not-combined, in a histogram). Thus, a dot-plot of the combination of Area X Modulation features may be a preferred strategy for specks identification, particularly for clinical purposes.

### “Bright Detail Similarity” Co-localizes Active Caspase-1 and Specks

Caspase-1 is synthesized as an inactive zymogen, pro-caspase-1 (45 KDa), and is activated by autoproteolytic cleavage after recruitment into the ASC-containing inflammasome complex (1). Autoproteolysis of caspase-1 results in removal of the N-terminal CARD domain thus originating an enzymatically active heterodimer comprised by large p20 (20 KDa) and small p10 (10 KDa) subunits (33–36). Because active caspase-1 mediates IL-1β and IL-18 activation and secretion, the analysis of cleaved caspase-1 is an important variable to assess in order to study inflammatory disorders where these cytokines might play a role. A simple method that has been extensively used to measure caspase-1 activity in cells employs the cell-permeable and non-cytotoxic probe FAM-FLICA™ (Fluorescent Labeled Inhibitor of Caspases), available from Immunochemistry Technologies (ICT) (37, 38). The FLICA reagent, FAM-YVAD-FMK, consists of the caspase-1 specific-inhibitor peptide sequence (YVAD) within a green fluorescent label called carboxyfluorecein (FAM) and a fluoromethylketone (fmk) moiety that covalently binds to all active caspase-1. Because FLICA probes become irreversibly coupled to the active enzyme, they are retained within the cell and the remaining green fluorescent signal is being considered a direct measure of caspase-1 activity present in the cell. Viable cells with inactive caspases do not bind FLICA probes. Unbound probes diffuse out of the cell and are washed away in subsequent washes. Cells that contain bound FLICA can be analyzed by flow cytometry or a fluorescence plate reader and therefore caspase-1 activity can be quantified. Indeed, it has been reported that FLICA staining is able to detect active caspase-1 at the site of the ASC focus by fluorescence microscopy (25, 39). Super-resolution microscopy of endogenous immunolabeled ASC and FLICA stained-THP-1 cells identified that the ASC ring surrounds caspase-1, therefore FLICA staining can be detected filling the central core of the ASC ring-like structure (27).

**Figure 4:**
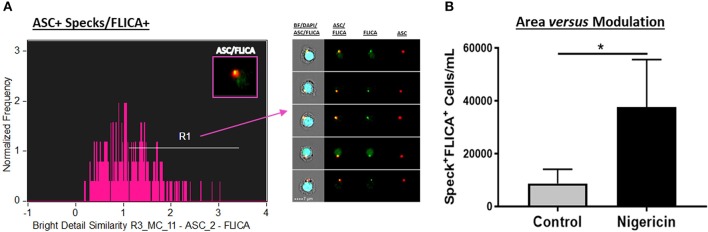
Co-localization of active caspase-1 with ASC Specks in Nigericin stimulated-CD14^+^ monocytes. **(A)** Histogram of Bright Detail Similarity R3_MC_11-ASC_2-FLICA inside Speck^+^ gate better identifies ASC specks with co-localized FLICA signal in in pam3csk4 (0.5 ug/mL, 3 h)- primed and Nigericin (3,5 uM, 1 h)-stimulated PBMCs. On the right side of the graph, representative images of Speck^+^FLICA^+^ monocytes were selected, showing, respectively: Composite image containing the fluorescences of ASC, FLICA, and DAPI with BF (brightfield), followed by Composite image of FLICA and ASC, and both fluorescences alone. Intensity fluorescence was augmented for the purpose of imagery, not affecting results and statistics. **(B)** Quantification of Speck^+^FLICA^+^ monocytes/mL in Control vs. Nigericin-stimulated cultures after Bright Detail Similarity R3_MC_11-ASC_2-FLICA Feature was applied in all data set. Numbers are the means ± SEM (*n* = 5). ^*^P < 0.05 when Mann-Whitney test was applied.

In order to combine the qualitative analysis of caspase-1 activation with a fast and statistically robust method, we have assessed ASC speck-bound active caspase-1 by ImageStream. For this purpose, upon stimulation, cells were incubated for 45–60 min, at 37°C, with the 1x FLICA probe as described in details in the materials and methods section, followed by Live/Dead and extracellular staining. To evaluate specks and caspase-1 co-localization, we applied a default application wizard of the IDEAS software called “co-localization” on the sample files. The co-localization wizard guides you through the process of measuring the co-localization of two probes with punctate staining (here, ASC and FLICA) in any population of cells you identify (in this case, inside the “Speck^+^ gate” that we previously created) by adding a histogram of “bright detail similarity R3” for the double positive population in the analysis area. The “bright detail similarity R3” feature evaluates the small bright image detail of FLICA and ASC and identifies the co-localization of these two probes in a defined region, in this case in specks. This feature is the log transformed Pearson's correlation coefficient of the localized bright spots with a radius of 3 pixels or less within the masked area in the two input images (description taken from the IDEAS User's Manual). Bright details of the two images are either correlated (in the same spatial location) or uncorrelated and the correlation coefficient varies between 0 (uncorrelated) and 1 (perfect correlation), not assuming negative values. The coefficient is log transformed to increase the dynamic range between {0, inf}. By visually checking the cells in the gallery, we drew a region to gate co-localized events of FLICA and ASC with values close to 1 and above. Figure 4A shows co-localized FLICA with ASC specks, and Nigericin-stimulated cells presented more activated caspase-1 in the center of the speck ring-like structure compared to non-stimulated ones (Figure 4B).

**Figure 5:**
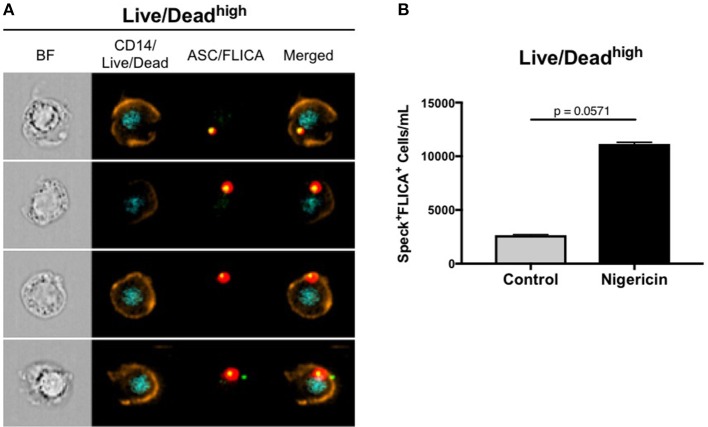
ImageStream can detect a Live/Dead AQUA^high^ subset of cells in different stages of pyroptosis. **(A)** Representative images of speck^+^FLICA^+^ monocytes inside the Live/Dead AQUA^high^ gate after pam3csk4 (0.5 ug/mL, 3 h) priming and Nigericin stimulation (3,5 uM, 1 h) were selected, showing, respectively: BF (brightfield), composite image containing the fluorescence of CD14 and Live/Dead AQUA, composite image of FLICA and ASC, followed by the merge of the two composites. Intensity fluorescence was augmented for the purpose of imagery, not affecting results, and statistics. **(B)** Quantification of Speck^+^FLICA^+^ monocytes/mL in control vs. nigericin-stimulated cultures after Area_Threshould(M11,11-ASC70) vs. Modulation_Morphology(M11,Ch11)_11-ASC dot-plot, followed by Bright Detail Similarity R3_MC_11-ASC_2-FLICA features were applied in all data. Numbers are the means ± SEM (*n* = 4). *P* = 0.0571 when Mann-Whitney test was applied.

As previously mentioned, besides pro-IL-1β and pro-IL-18, active caspase-1 also cleaves gasdermin D (GSDMD). Cleaved-GSDMD oligomerizes in the plasmatic membrane to form pores that permeabilize the cell, subsequently leading to a complete osmotic lysis, a process of cell death that is known as pyroptosis (40). In fact, we observed permeabilized cells that allowed for Live/Dead AQUA intracellular staining, presenting ASC speck formation with active caspase-1 (Figure 5A), therefore being distinct from viable cells containing specks with intact plasmatic membrane (Live/Dead AQUA^low^). We consider that this Live/Dead AQUA^high^ subset represents cells in late stages of pyroptosis, but still preserving the inflammasome complex intracellularly. Accordingly, although not reached statistical significance, higher amounts of monocytes with this phenotype was observed in nigericin-stimulated cultures than controls (Figure 5B), consistent with Nigericin-induced caspase-1-mediated cell death. Therefore, due to the high throughput associated with the spatial measurements obtained with MIFC, our results demonstrate how Imagestream is a powerful technique to evaluate reliably caspase-1 activity and downstream events, shuc as cells in different stages of pyroptosis.

### Increased FLICA^+^ASC Speck Formation in Clinical Samples

**Figure 6:**
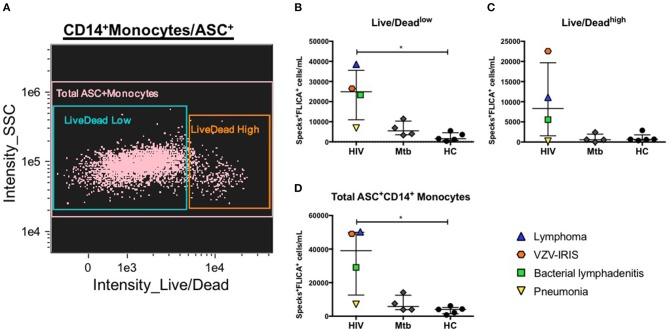
Evaluation of canonical inflammasome activation in patient samples. **(A)** Representative 2-parameter dot-plot fluorescence of Live/Dead (L/D–channel 8) vs. side scatter (SSC–ch12) applied after population of interest was defined as follow: single cells, focused cells, monocyte-like population (based on area and SSC), HLA-DR^+^, and DUMP^−^ cells and finally CD14^+^ASC^+^ cells. Speck^+^FLICA^+^ monocytes/mL were then quantified in PBMCs from healthy controls (HC) and from patients after Area_Threshould (M11,11-ASC70) vs. Modulation_Morphology(M11,Ch11)_11-ASC dot-plot, followed by Bright Detail Similarity R3_MC_11-ASC_2-FLICA features were applied in all data inside Live/Dead AQUA^low^ **(B)**, Live/Dead AQUA^high^ **(C)**, and total CD14^+^ASC^+^ cells **(D)** as defined in **(A)**. ^*^P < 0.05 when Mann-Whitney test was applied.

We previously described how to better identify catalytic active ASC speck formation by using an ultimate positive control that were cells from healthy individuals stimulated with PAM3 plus Nigericin, an NLRP3 inflammasome agonist. As a proof of concept of the application of this method in clinical conditions, we investigated the presence of caspase-1-containing specks in two distinct groups of patients. Because both HIV and *Mycobacterium tuberculosis* (Mtb) have been shown to activate the NLRP3 inflammasome (41, 42), and it has been reported that polymorphisms or activation of some inflammasome genes can affect Mtb infectious and HIV-related inflammatory conditions, in humans (43–47), we decided to investigate inflammasome activation in four patients presenting active pulmonary tuberculosis and four HIV^+^ individuals with distinct HIV-related complications: one viremic anti-retroviral therapy (ART) naïve patient with lymphoma, and three patients with suppressed HIV viremia on ART but with acute events, one with a Staphylococcal axillary lymphadenitis, the second one with community acquired pneumonia and the last one with immune reconstitution inflammatory syndrome (IRIS) related to Varicella-zoster virus (VZV) co-infection (VZV-IRIS). Applying the same features identified as best discriminators of ASC speck formation found in our nigericin-treated control analysis, we observed that HIV^+^ patients, in general, presented elevated numbers of monocytes with spontaneous FLICA^+^ASC speck formation compared with healthy volunteers, independently of the viability state (Live/Dead staining low or high fluorescence) of the cells (Figures 6A–D). Interestingly, the patient with pneumonia scored low numbers of monocytes with specks, similar to the Mtb group, suggesting that localized inflammatory pathologies may not always be reflected in circulating blood monocytes. Thus, our results demonstrate that ImageStream is sensitive enough to capture *in vivo* inflammasome-activated cells, distinguishing different inflammatory conditions from healthy individuals.

## Discussion and Conclusions

ASC recruitment is a key event for canonical inflammasome activation, not only because it links activated inflammasome sensors to the effector molecule pro-caspase-1, but also provides the conformational changes that are crucial to drive caspase-1 autoproteolysis and subsequent inflammatory cytokine production. Here, we demonstrated that multispectral imaging flow cytometry provides a very simple, fast and reliable method to detect ASC specks in human monocytes, in a quantitative and qualitative manner. Following wizard instructions on IDEAS software, we found at least 6 good features that provide an efficient strategy to discriminate between spread ASC pixels and cytosolic translocation of ASC molecules into aggregates, by using a positive control for NLRP3 inflammasome activation.

Furthermore, we also aimed to determine what feature performs best in discriminating the specks. One way to compare how well a Feature can distinguish between spread ASC and cells containing ASC specks would be to use the Rd score. As previously mentioned, the higher the Rd, the greater the separation between the two sample populations. By analyzing the histograms that combine the features with the highest Rd, we observed that despite small differences in their Rd values, virtually all the first 6 features (with Rd scores >2) clearly distinguished diffuse ASC and ASC specks, thus demonstrating that we have different options that are able to identify canonical inflammasome activation by using ImageStream. Interestingly, half of the top 6 features screened by feature finder for our data set belong to the “size” category (“Perimeter,” “area,” and “minor axis”). Since the most striking characteristic of ASC redistribution upon inflammasome activation is that it varies from a diffuse form to a condensed single spot, it is reasonable to understand why features that are able to measure changes in the dimension of the ASC staining would be suitable for discriminating the specks. Simultaneously, the signal coming from a Speck is also brighter than the signal of the scattered ASC pixels in resting cells. This is measured in IDEAS as an increase of the intensity and through changes in the texture/contrast of the image. On this note, this explains why the other half of the 6 best features were “Texture” features (“H features”: “homogeneity” and “entropy”; and “Modulation”), since a notable characteristic of texture features is that they identify intensity variations in the image.

We applied the best suggested features or feature combinations to our population of interest and carefully adjusted the gates in accordance with the correspondent imagery. We observed that some features or feature combinations were more permissive to false-positive events, because they did not take into consideration both of the fundamental aspects of speck formation simultaneously: variation of intensity and size of the ASC signal. Interestingly, among all the best features identified by “feature finder” with higher Rd scores, modulation alone did a better job (data not shown). This feature points out contrast of pixels with background, better contrast gives higher scores and this does not occur when ASC is diffuse. However, we observed that the combination of this “texture feature” with a “size feature” (that in this study “area” had the best score), is the best strategy to achieve the most accurate detection of specks, covering both essential characteristics of the speck.

Our findings are in accordance with a recently developed method to quantify specks by conventional flow cytometry, in which the same principle was applied to measure ASC specks. The authors evaluated the height (equal to the intensity of the signal) in combination with the width (equivalent to the size of the signal) of the ASC fluorescence signal pulse to detect ASC specks (28, 29). While it is important to note that “intensity” is also a feature value that can be extracted in IDEAS, this feature had inferior performance compared with “modulation” to identify speck formation (in our dataset achieved a good Rd score of 1.5 to discriminate specks vs. difuse ASC). An advantage of applying “modulation” over “intensity” is that the first feature identifies the regional variation of intensity of the pixels, thus adding the granularity or complexity of an image as a parameter, while “intensity” measures the overall intensity of the distributed pixels in the cells. Additionally, “modulation” is a feature based on the spatial distribution of the signal, therefore not available in conventional flow cytometry method, thus making the analysis by MIFC more complete by not just relying in only one parameter measurement but also having the imagery to visually confirm the presence of specks.

Importantly, we also demonstrated that ImageStream was able to detect the catalytically active form of caspase-1 associated with specks, showing that they were functionally competent. This was confirmed by the fact that Nigericin-treated samples presented increased numbers of monocytes containing active specks than controls, by having higher co-localization score of ASC and FLICA. This is another good example of advantages of using MIFC over traditional flow cytometry, as the spatial information obtained by MIFC is required to measure co-localization. We also demonstrated that this method was able to assess cells that underwent caspase-1 activation in different stages of pyroptosis. Although cells might release the inflammasome complex to the extracellular milieu at the latest stages of cell death (48, 49), we were still able to account for the inflammasome-activated cells in different stages of GSDMD-mediated membrane pore formation by considering the total amount of cells containing specks with FLICA, regardless of their viability status.

Finally, we confirmed that the use of ImageStream is a sensitive method to evaluate circulating inflammasome activation in clinical samples. Here, we were able to discriminate the amount of monocytes with FLICA^+^ speck formation in patients suffering with distinct HIV-related inflammatory complications (i.e., lymphoma, bacterial lymphadenitis, VZV-IRIS) from healthy volunteers. Accordingly, we have also recently reported aberrant inflammasome/caspase-1 activation in HIV-1^+^ patients with extreme CD4^+^ T cell decline despite ART-mediated viral suppression, as one of the possible mechanisms explaining this rare condition, by using the method described in this study (47). We also observed discrepant results between an HIV^+^ patient with pneumonia who had a lower score such as Mtb-infected individuals and other HIV^+^ patients with systemic inflammatory disorders. We believe such differences might be due to measuring inflammasome-activated circulating blood monocytes instead of assessing inflammasome activation in restricted lung areas affected by Mtb or *Staphylococcus aureus*. Interestingly, the observation of cells containing specks and a high Live/Dead AQUA phenotype in patient samples suggests that caspase-1-activated monocytes underwent pyroptosis and those cells can be captured by ImageStream. This is particularly important, since free ASC aggregates released from pyroptotic cells to the extracellular space have been proposed as new “damage signals,” being able to either directly target extracellular pro-caspase-1 and/or induce phagolysosomal damage, and consequently NLRP3 activation, in neighborhood cells (48, 49). This leads to a positive feedback loop in IL-1β/IL-18 maturation and secretion, thus affecting many diseases where inflammasome play a relevant role. Regardless of the Live/Dead status, however, the same differences among the groups of patients were observed, thus confirming low inflammasome activation detected inside the Live/Dead AQUA^low^ subset for some patients was not due to pyroptosis. For distinct experimental purposes, however, when cells need to be stimulated *in vitro*, preventing pyroptosis induction downstream of caspase-1 activation by the addition of the selective caspase-1 inhibitor Y-VAD in cell cultures must be considered, although in that case caspase-1 activity itself cannot be measured by FLICA staining.

In summary, our observations highlight the advantages of having the imagery of the cells to confirm the choice of the algorithms for ASC specks identification and determine their functional state (i.e., active caspase-1 associated with the speck). The ability of ImageStream to collect images of large numbers of cells with high magnification renders it to be a powerful tool for both experimental and diagnostic purposes, since inflammasomes have been implicated in an extensive range of chronic inflammatory conditions. Moreover, besides distinct well-known therapies blocking IL-1β activity, many scientific groups are trying to develop therapeutic approaches that target various components of the inflammasomes, such as NLRP3, ASC and caspase-1, in an attempt to simultaneously block all downstream effects of the inflammasome activation (50–52). In this sense, data described here demonstrate how multispectral imaging flow cytometry can be an efficient tool for high throughput screening of drug targets that modulate inflammasome activation.

## Ethics Statement

Blood samples were obtained from healthy donors on the NCI, NIH IRB-approved protocol 99-CC-0168 and de-identified prior to distribution. All subjects gave written informed consent in accordance with the Declaration of Helsinki. ClinicalTrials.gov Identifier: NCT00001846 (National Institutes of Health, Bethesda, MD, USA).

## Author Contributions

SLL, VMD, C-SW, and IS were involved in conception and design of the study and methodology. SLL performed experiments and acquisition of data. SLL and VMD performed data analysis. SLL, VMD, and IS were involved in manuscript writing and review. All authors have read and approved the final manuscript.

### Conflict of Interest Statement

The authors declare that the research was conducted in the absence of any commercial or financial relationships that could be construed as a potential conflict of interest.
